# Patient Perceptions of Embryo Visualisation and Ultrasound-Guided Embryo Transfer During IVF: A Descriptive Observational Study

**DOI:** 10.3390/jpm15080374

**Published:** 2025-08-13

**Authors:** Giorgio Maria Baldini, Dario Lot, Antonio Malvasi, Antonio Simone Laganà, Angelo Alessandro Marino, Domenico Baldini, Giuseppe Trojano

**Affiliations:** 1 Obstetrics and Gynecology Unit, Department of Biomedical Sciences and Human Oncology, University of Bari “Aldo Moro”, 70121 Bari, Italy; gbaldini97@gmail.com (G.M.B.); antoniomalvasi@gmail.com (A.M.); 2 IVF Center, Momo Fertilife, 76011 Bisceglie, Italy; dariolot92@gmail.com; 3 Unit of Obstetrics and Gynecology “Paolo Giacone” Hospital, Department of Health Promotion, Mother and Child Care, Internal Medicine and Medical Specialities (PROMISE), University of Palermo, 90135 Palermo, Italy; antoniosimone.lagana@unipa.it; 4 Reproductive Medicine Unit, ANDROS Day Surgery Clinic, 90121 Palermo, Italy; angelo.marino@clinicaandros.it; 5 Department of Maternal and Child Health, Madonna delle Grazie Hospital, 75100 Matera, Italy; giuseppe.trojano@asmbasilicata.it

**Keywords:** infertility, in vitro fertilisation, ultrasound-guided embryo transfer, patient satisfaction, patient-centred care, emotional well-being, doctor continuity, embryo viewing

## Abstract

**Objective:** To evaluate patient perceptions regarding ultrasound-guided embryo transfer, visualisation of embryos prior to transfer, and continuity of care with the same physician during in vitro fertilisation (IVF) treatments. **Setting:** Between January and September 2023, this study was conducted at the IVF MOMO’ FertiLIFE centre in Bisceglie, Italy. **Design:** Descriptive and observational study based on an anonymous survey administered to IVF patients at the time of embryo transfer. The goal was to assess the subjective emotional and psychological response to selected procedural elements of the embryo transfer process. **Participants:** Out of 284 distributed questionnaires, 200 were included in the final analysis. Inclusion required fully completed responses. Questionnaires with incomplete, unclear answers or patient refusal were excluded. The study group was compared with the general IVF patient population treated at the centre over the past 5 years to ensure representativeness. **Methods:** Patients completed a structured questionnaire using a five-point Likert scale. Statistical analysis included descriptive statistics, Spearman’s rank correlation, Friedman test, and exploratory factor analysis. Ethical approval was obtained (CELFer no. 07/2021), and all participants provided written informed consent. **Results:** The majority of patients reported a heightened sense of calm and reassurance during ultrasound-guided embryo transfer. Viewing embryos on a monitor before transfer was also positively perceived. A strong preference emerged for continuity of care with the same physician throughout the IVF process. While this study did not assess objective stress levels or clinical outcomes, the findings highlight the psychological comfort associated with these patient-centred practices. **Limitations:** This single-centre study is based on self-reported data and lacks objective assessments of psychological well-being. Therefore, results reflect personal perceptions rather than measurable clinical outcomes. Broader, multicentre research using validated psychological tools is needed to confirm and expand these findings. Furthermore, the questionnaire used in this study was developed internally and not validated externally with standardised psychometric instruments. **Conclusions:** This study provides insight into IVF patients’ subjective experiences, emphasising the perceived emotional benefits of specific procedural and relational aspects of care. These findings support the integration of patient-centred strategies—such as visual engagement and physician continuity—into routine IVF practice to enhance overall patient well-being.

## 1. Introduction

Infertility and related treatments are associated with a significant psychological and physical burden [1,2,3,4,5]. As patient-centred care addresses the needs and values of patients, it may be beneficial for infertile individuals [6]. The centrality of the patient is one of the key elements of high-quality care [4,7,8,9,10,11,12,13]. Many daily activities of patients focused on the possibility of pregnancy involve routine visits to doctors, sample collection, frequent tests, medication intake, and even surgical procedures, often with minimal or no shared decision-making. In the context of in vitro fertilisation (IVF), embryo transfer is a critical stage, during which embryos are transferred into the patient’s uterus to facilitate conception. While the success of this procedure depends on numerous biological, clinical, and technical factors, patients’ emotional and psychological experience plays a key role in their overall well-being and treatment outcome [4]. Poor doctor–patient communication can be a predictive factor for treatment abandonment, contributing to the high costs associated with infertility treatment [13]. Consequently, the medical staff caring for these patients, particularly doctors, are often subject to high expectations, as they are seen as problem solvers. The doctor and the couple form a relational triangle [14,15]. The implementation of ultrasound-guided embryo transfer has improved the accuracy and safety of the procedure, enabling more precise embryo placement [16,17]. Nevertheless, its influence on patients’ perceptions and overall satisfaction continues to be a subject of investigation. Infertile couples often experience various aspects of care as problematic [18]. Thus, infertility treatment is typically described as a process that requires both time and energy [19]. There is strong evidence that doctor–patient communication is a good predictor of patient compliance, treatment adherence, clinical outcomes, and overall patient satisfaction [7,8,9,10]. How the doctor and patient relate to each other can create a shared meaning between these two parties [8]. Even indirect communication can yield benefits in specific treatments. The shared use of medical devices, such as transabdominal ultrasound for ultrasound-guided embryo transfer or the viewing of embryo images on a screen remotely connected to a microscope before transfer, may be perceived by patients as reducing the anxiety associated with this stage, which is usually driven by a lack of knowledge and participation in decision-making [20]. Doctors must provide professional assistance in the couple’s decision-making process, understand the most advanced reproductive technologies in depth, and be empathetic, caring, sensitive, and genuinely involved [21]. The use of these devices may support patient understanding and involvement. Although many studies have investigated the determinants of optimal patient experiences or satisfaction with fertility care [22,23,24], most of the literature has focused on patients’ demographic, medical, or psychological characteristics, such as social class, infertility duration, or self-esteem [25,26,27]. However, this study aims to examine patient satisfaction regarding two aspects of the embryo transfer process: the experience of undergoing ultrasound-guided transfer and the opportunity to observe the embryos on a screen before transfer. While both approaches are increasingly used to improve transfer effectiveness, how they influence patient perceptions and overall treatment experience is still unclear. Another important factor from our study is patients’ preference for continuous care from the same physician [27]. The possibility of being treated by the same professional throughout the different stages of the therapeutic process appears to increase patients’ sense of security and trust, positively impacting their psychological well-being [28,29,30]. Through a direct survey of patients treated at our centre, we gathered information about their satisfaction with these techniques, the emotions associated with their use, and their preference for continuous care with the same doctor. This study may contribute to understanding the importance of the psychological aspect of IVF, improving patient experiences and, potentially, overall treatment outcomes.

## 2. Materials and Methods

### 2.1. Population and Study Design

From January 2023 to September 2023, we surveyed patients who underwent an embryo transfer at the IVF MOMO’ FertiLIFE centre in Bisceglie, Italy. All couples recruited were awaiting a fresh or cryopreserved embryo transfer. There were no specific inclusion or exclusion criteria. This was a descriptive and observational study. Epidemiological characteristics, infertility duration, education, and occupation were compared with statistical data regarding patient influx at our centre over the past 5 years. This evaluation was obtained using the MediITEX management software package 4.0.1.0 (CRITEX GmbH, Regensburg, Germany). Written informed consent was obtained from all patients. Participation in the survey was voluntary and anonymous. This study was conducted in accordance with the ethical criteria of the Declaration of Helsinki. The positive opinion of the local ethics committee (CELFer no. 07/2021) was obtained. Clinical trial number: not applicable.

Anonymity was ensured as the questionnaire contained no identifying information about the respondent. The completed questionnaires were then placed into a sealed box by the patients.

### 2.2. Data Collection

Out of 284 original questionnaires, 200 were included in the final analysis. A total of 84 questionnaires were excluded: 43 due to incompleteness, 28 due to unclear answers (e.g., multiple options selected where only one was requested), and 13 because the patient declined to complete the survey.

These 200 patients were then compared with a control group in terms of epidemiological characteristics. The control group included all patients who attended the IVF MOMO’ FertiLIFE centre over the past 5 years. This comparison aimed to verify the representativeness of the study group in relation to the overall patient population.

The statements were evaluated using a 5-point Likert scale [31]. To identify the determinants related to optimal patient experiences cited in the questions, data were collected on eight specific questions:

1. Do you prefer always interacting with the same doctor rather than seeing a different doctor each time?
2. Would you be more satisfied if the gynaecologist or embryologist, besides providing the embryo culture results, showed you the embryo images on a screen in the transfer room in real time before the transfer?
3. Does the ultrasound during ultrasound-guided transfer cause you discomfort?
4. Does the performance of ultrasound-guided embryo transfer make you feel more secure?
5. Does viewing the embryos on a screen and seeing the ultrasound-guided transfer on another screen help you face the transfer more calmly?
6. Do you believe that ultrasound-guided transfer and viewing your embryos during the procedure may infringe on your privacy?
7. Do you consider it important for your partner to be present during the transfer?
8. Do you think the midwife’s presence in the transfer room, in addition to the possible presence of your partner, infringes on your privacy?

**Figure 1:**
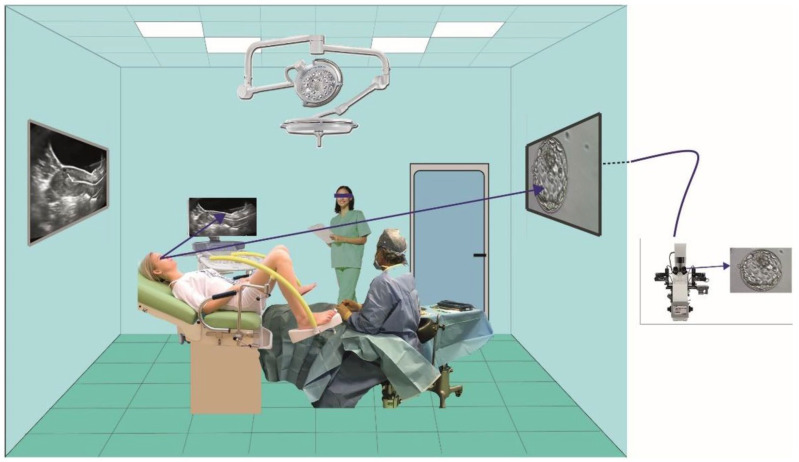
The figure shows the patient about to undergo the transfer and she can see both the image of the embryo to be transferred (which comes from the microscope) and the screen with the ultrasound image.

The transfer room was set up so the physician could view the ultrasound image and the patient could view the embryos to be transferred (Figure 1).

During the questionnaire’s completion, the patient was left alone in the transfer room to ensure no external influence could affect her responses. The completed questionnaire was then placed by the patient into a sealed box, ensuring that she could verify the anonymity of her answers. The same researcher handed all questionnaires to the patient.

### 2.3. Patient’s Experience

Before distributing the questionnaires, a pilot test (conducted with a small sample of 12 patients) was carried out to identify any difficulties in interpreting the questions. The results from the pilot group indicated that the questions were easily understandable, and thus, the test was confirmed. Each question was associated with five possible responses according to the Likert scale to ensure that responses were more standardised and less susceptible to personal interpretation variations. The following 5-point responses were used:

1. Very much;
2. Quite a bit;
3. Moderately;
4. A little;
5. Not at all.

We used a 5-point Likert scale [31] for data collection to effectively measure patients’ opinions, feelings, and perceptions regarding different experiences during in vitro fertilisation treatment. The Likert scale is widely used in psychological and social studies to assess attitudes and opinions on subjective variables. It is particularly suitable for studies where responses are not dichotomous but span a range of intensity. In particular, the Likert scale offers the following advantages:

- Detection of nuances in responses;
- Ease of understanding and standardisation;
- Quantification of subjective experiences;
- Detection of variability in experiences;
- Validity and reliability.

We also attempted to minimise social desirability bias through the following:

- **Strict anonymity:** Ensuring that responses were completely anonymous and that no one (not even the centre staff) could associate reactions with a specific patient helped reduce the risk of answers being influenced by the desire to please the researchers or medical staff.
- **Confidentiality statement:** We strengthened communication regarding the confidentiality of the information, explaining that responses would not affect the treatment or care received. Thus, we reduced bias related to fear of external judgment.

To avoid acquiescence bias, we included negatively worded questions to balance the positive and negative items and control questions. In addition to the pilot test, we conducted an internal validation of the questionnaire. Reliability was confirmed with a Cronbach’s alpha of 0.74, which is considered acceptable. Furthermore, an exploratory factor analysis (EFA) was carried out, revealing that the first four components had eigenvalues > 1, explaining 56.2% of the total variance. These analyses suggest that the questionnaire has an acceptable internal structure and measures distinct constructs. However, we acknowledge that the questionnaire has not been validated against external psychometric tools, which represents a limitation of the present study.

### 2.4. Organisational Determinants

To consider potential organisational determinants, a group of experts (gynaecologists, a psychologist, a fertility nurse, and a quality manager) discussed which organisational aspects could influence the patients’ experiences. The group’s decision-making process was supported by evidence from the general literature and the fertility-specific literature [26,27,28,29]. Organisational determinants selected included critical points to ensure anonymity, information about the embryo viewing process, explanation of the ultrasound-guided transfer catheter, placement of responses into the sealed box, and always having the same doctor [28] to avoid different empathetic processes.

### 2.5. Statistical Analysis

We used Prism 10 and SPSS 26 for all statistical analyses. Given the annual number of treatments at our centre (600 cycles), with a margin of error of 5%, a 95% confidence level, and a standard deviation of 25%, the sample size was calculated to be 195 per group (we recruited 200). We chose a 5% margin of error to ensure that the results obtained from the 200-patient sample were representative of the target population, with a 95% probability that the observed results would fall within a ±5% range of the values that would have been obtained if the entire IVF centre patient population had been surveyed. Mean dimension scores were calculated only for participants who completed the survey. These scores could range from 1 to 5 (according to the direction of the Likert scale).

### 2.6. Descriptive Analysis of Responses

First, we performed a descriptive analysis to understand the distribution of responses for each of the eight questions. Descriptive statistics included the mean, median, standard deviation, percentages of responses for each level of the Likert scale, and significance testing using the chi-square test.

### 2.7. Correlation Analysis Between Questions and Significance

We used Spearman’s correlation tests to assess whether there was a monotonic relationship between responses to two or more questions on the Likert scale. Pearson’s correlation is suitable for continuous variables that follow a normal distribution (i.e., linear data without severe distortion). However, Spearman’s correlation is more appropriate for variables that are not normally distributed or for ordinal data (like the Likert scale).

### 2.8. Analysis of Differences Between Responses to Multiple Questions

Using the Friedman test, we aimed to assess whether there were significant differences in responses across all eight questions (e.g., whether some questions received significantly higher responses than others). This non-parametric test compares the means of dependent groups (i.e., when responses are “paired,” as in repeated measures across multiple questions).

### 2.9. Reliability Analysis of the Scale

After adjusting responses with the reverse direction, Cronbach’s alpha was calculated to test internal reliability. A Cronbach’s alpha value above 0.7 is generally considered acceptable, while values above 0.8 or 0.9 are considered good.

### 2.10. Factor Analysis

A factor analysis was conducted to identify whether the questions clustered into underlying factors (e.g., groups of questions measuring similar concepts). Specifically, an **exploratory factor analysis (EFA)** was used to identify the number of factors explaining the variance in the data, with the eight questions naturally grouped into underlying categories.

## 3. Results

### 3.1. Organisational and Patient Determinants

Table 1 provides information on all the initial determinants. The study group consisted of 200 patients, while the control group was derived from a sample of 3174 patients who underwent fresh and frozen embryo transfers at our centre over the last 5 years. The controlled epidemiological data included age, marital status, duration of infertility, education level, and occupation. No significant differences were observed when the Student’s *t*-test was evaluated. This allowed for us to conclude that the study group closely resembled the average condition of patients who seek ART (Assisted Reproductive Technology) procedures at our centre, particularly embryo transfer. This evaluation is important as it suggests that the responses of the study group should reflect those of patients typically undergoing embryo transfer at our centre.

Table 2 provides a detailed summary of the questions and answers, highlighting responses on the Likert scale, percentages, means, medians, standard deviations, and the chi-square index. It is evident from the reactions that patients expressed a preference for continuity of care, which they associated with greater trust and emotional comfort (84% + 21% in favour vs. 3% against), and this result was statistically significant according to the chi-square test (*p* < 0.0001). The preference for using guided embryo transfer and viewing the embryos during the procedure was particularly noticeable in responses to questions 2-4-5, with percentages of 85%, 85%, and 91.5%, respectively. Chi-square was also significant here. Question 3 explored whether, despite being satisfied with these techniques, they caused any discomfort. The response was overwhelmingly substantial, with 86% of patients reporting no discomfort. Chi-square also showed significance. Whether these measures promoted a sense of calm during the procedure (Question 5) had a positive response of 91.5%, with chi-square significant. Analysing Questions 7 and 8, it is quite remarkable that 28.5% and 6.5% of patients did not perceive the presence of their partner as necessary, while it was indicative that the presence of the midwife did not interfere with privacy (97% with chi-square significant).

We also sought to verify whether some questions, particularly Questions 2, 3, and 5, might exhibit a correlation and statistical significance. To this end, we used Spearman’s rank correlation (Table 3) to assess associations between responses. The results revealed weak correlations, with all correlation coefficients close to zero (r = 0.0073 for Q2, r = 0.0141 for Q4, and r = 0.0324 for Q5), and *p*-values well above 0.05, indicating that no statistically significant correlation was found between the questions analysed. These findings suggest that patient perceptions of these elements were largely independent.

To analyse differences between responses across multiple questions, we performed the Kruskal–Wallis statistical test (Table 4). The results clearly showed significant differences (*p* < 0.0001), indicating that the questions were designed to assess different aspects of the embryo transfer experience. Since the *p*-value is below 0.05, we can conclude that there are significant differences in the responses to the eight questions.

### 3.2. Reliability Analysis of the Scale

Cronbach’s alpha was calculated to test internal reliability after adjusting responses with reversed scoring. The resulting Cronbach’s alpha was 0.74, generally considered acceptable (above 0.7). To assess whether the set of questions yielded similar concepts, exploratory factor analysis (EFA) was performed (Table 5). The eigenvalues represent the variance explained by each component (factor). Generally, a higher eigenvalue indicates that the component explains a greater portion of the variance in the data. An eigenvalue greater than 1 indicates that the component has sufficient significance to be retained in the analysis. The eigenvalues show that the first four components have values greater than 1, so they should be considered significant and retain a substantial portion of the variance in the data. The eigenvalues for component 5 (0.967), component 6 (0.943), component 7 (0.884), and component 8 (0.710) are below 1, so based on the Kaiser criterion, they could be considered less relevant to the analysis. The percentage of variance explained by each component indicates how much each factor contributes to explaining the total variance in the question responses. Cumulative variance shows the total variance explained by the factors considered up to that point. Cumulative variance indicates the percentage of total variance explained by the factors subsequently extracted. Overall, the first four components explain 56.201% of the total variance, while the first five explain 68.288%. Based on the Kaiser rule (eigenvalues > 1) and the percentage of variance explained, the first four questions appear to be the most significant. However, these four only explain about 56.2% of the total variance, which is a good portion but incomplete. So, we decided to add a larger portion of the variance (e.g., more than 60%) by including the fifth question (which brings the cumulative variance to 68.3%). This way, we can give a more detailed description of the variables. Therefore, we can conclude that the cumulative variance grows progressively, reaching 100% with eight components, but the first four or five generally provide the greatest informative value.

## 4. Discussion

The treatment of infertility, particularly through techniques such as in vitro fertilisation (IVF), extends beyond strictly medical and biological concerns, including the empathic relation with the nurses [32,33], encompassing significant psychological and social dimensions [34,35,36]. Fertility treatments often involve a demanding journey for patients, characterised not only by physical discomfort but also by substantial emotional stress, anxiety, and feelings of inadequacy, which can adversely affect their quality of life as well as treatment outcomes [37,38]. This study explored patients’ psychological responses to the adoption of innovative practices, such as ultrasound-guided embryo transfer and embryo visualisation, aiming to understand how these practices influence their experience during IVF treatment.

### 4.1. Patient-Centred Care and the Doctor–Patient Relationship

Patient-centred care is a core principle of modern healthcare [38], and this study emphasises how the doctor–patient relationship, particularly in the context of assisted reproduction, may influence patient perceptions of satisfaction and comfort. A key finding was that 84% of participants preferred to be treated by the same physician throughout their care. This suggests that patients highly value continuity of care, as it fosters the development of trust and rapport with their doctor. Effective, empathetic communication, characterised by a genuine willingness to listen to patients’ concerns, is crucial for alleviating the anxiety and fear associated with treatment [1]. Familiarity with the physician also strengthens trust in their professional competence and helps reduce the perception of the procedure as impersonal or invasive [39]. Therefore, the continuity of the doctor–patient relationship seems to play an important role in improving the psychological perception of the treatment, as patients reported feeling more secure. Patients are more likely to disclose information, and medical management is more likely to be adapted to the patient’s personal needs. Increased patient satisfaction may also be associated with enhanced “optimism” regarding health outcomes [40]. Continuity of care is also associated with the perception that the physician has become more responsive [41]. A deeper analysis of the responses revealed the vulnerability of patients undergoing treatment, as, despite sufficiently comprehensive counselling, the description of the treatment leaves many questions unanswered, mostly related to lack of knowledge. Therefore, empathy with the doctor [29] and using devices that help patients understand and physically experience the procedures being performed can make this phase of treatment more reassuring. Unfortunately, there has been a generational and organisational shift in IVF treatments that increasingly sacrifices the doctor–patient relationship, moving toward a more commercial model that is less concerned with healthcare. This phase of the doctor–patient relationship must be restored through stable relationships with the doctor and appropriate aids that reduce the stress to which patients are subjected.

### 4.2. Ultrasound-Guided Transfer: Precision and Psychological Comfort

Ultrasound-guided embryo transfer is a practice that allows for greater precision in implantation [42,43], improving not only the effectiveness of the treatment but also the experience of the patients. The study data indicate that 86% of participants did not experience discomfort during the ultrasound-guided procedure, with 85% stating that they felt more secure due to this technique. This finding is of considerable significance, as the perceived invasiveness of a medical procedure can profoundly impact patients’ psychological well-being. Incorporating ultrasound guidance during embryo transfer alleviates uncertainty and enhances the transparency of the procedure. Moreover, it enables patients to feel a greater sense of “participation” in the process, augmenting their perceived control over their fertility and treatment outcomes. In this regard, the precision afforded by ultrasound has not only clinical but also psychological benefits. It ensures optimal embryo placement within the uterus, thereby reducing the likelihood of procedural errors and improving success rates [44]. Beyond the clinical advantages, the perception of a targeted approach appears to positively influence patients’ emotional states, as the assurance that the procedure is being conducted with the highest level of expertise and precision helps to mitigate anxiety and the fear of failure. This result supports the theory that greater control over medical procedures may support patients in feeling less stressed and may contribute to a more positive treatment experience [5,6]. Performing ultrasound-guided transfer and viewing the “white spot” on the ultrasound screen after the transfer generally alleviates the anxiety associated with the embryo potentially “disappearing” or even “leaving” the genital tract. Additionally, some patients clearly understand, as explained in transfer counselling, that the goal is to deposit the embryo in a more suitable area to avoid pregnancies in unsuitable locations.

### 4.3. Privacy and Confidentiality: A Delicate Balance

Protecting privacy and confidentiality during in vitro fertilisation (IVF) is a fundamental component of patients’ psychological well-being. While certain medical procedures may be perceived as invasive, patients generally do not view ultrasound-guided embryo transfer and visualisation as infringements on their privacy. Indeed, 98.5% of participants indicated that they did not feel uncomfortable with the privacy aspects of the procedure. This finding underscores the importance of medical practices that emphasise transparency and respect for patient dignity, as such approaches can reduce the perception of invasiveness and contribute to a more comfortable and secure environment. Safeguarding patient privacy during procedures and providing a private and protected space is essential to preventing feelings of shame or embarrassment, which can adversely affect emotional well-being and treatment outcomes [1,2,3,4,5]. Thoughtful management of physical spaces, integrating anonymisation practices (such as securing survey responses), and a sensitivity to patients’ privacy concerns are key to creating a positive psychological environment, ultimately enhancing the overall patient experience.

### 4.4. Embryo Visualisation: The Effect of Visual Transparency

Real-time embryo visualisation during the in vitro fertilisation (IVF) process has been widely endorsed by patients. Approximately 85% of participants expressed greater satisfaction when observing their embryos directly, indicating that active engagement in the treatment process enhances the psychological experience. The ability to visually perceive the result of the procedure facilitates stronger emotional involvement and fosters a sense of agency, thereby mitigating the emotional detachment often observed in fertility treatments. Furthermore, embryo visualisation reduces uncertainty related to both the procedural aspects and the anticipated outcomes. Patients view this opportunity as an empowering experience, which cultivates a more profound emotional and psychological connection to the treatment, thereby enhancing overall treatment satisfaction and well-being. Previous studies have suggested that visual techniques in medical practices can positively affect patients’ psychological health, reducing anxiety and stress [7,8]. Embryo visualisation, therefore, not only improves the transparency of the treatment but also positively influences patient satisfaction and their perception of control over their fertility. However, it is important to acknowledge that, for some patients, detailed embryo visualisation may lead to increased anxiety or self-blame, particularly when embryos are perceived as low quality. Excessive attention to morphological details could heighten stress levels rather than alleviate them. Therefore, it is essential for medical staff to provide clear, empathetic communication and psychological support when presenting embryonic images, ensuring patients receive adequate guidance and emotional reassurance.

### 4.5. Partner Presence: Emotional Support and Social Context

The presence of the partner during embryo transfer is a controversial issue. Most patients (59.5%) stated that the emotional support of their partner is important, but 28.5% indicated that the partner’s presence is not essential. This finding suggests that while many patients desire the emotional support of their partner, others may prefer to undergo the procedure in a more intimate context or without the presence of a third person. Patient perceptions are highly individualised, and personalising treatment to account for each patient’s preferences, such as the desire to have their partner present, could further enhance the psychological experience. This is particularly important, as emotional support from a partner can significantly contribute to the patient’s well-being during treatment. However, it is crucial to recognise that perceived pressure or intrusion by the partner may have the opposite effect, potentially increasing anxiety and diminishing the patient’s sense of control. As such, tailoring the approach to respect individual preferences regarding partner involvement is essential for optimising psychological outcomes and overall treatment satisfaction.

### 4.6. Reliability of Results and Correlations

From a statistical standpoint, the Spearman correlation analysis revealed significant associations between responses related to the patient’s experience with ultrasound-guided transfer and embryo visualisation. The strong correlation between these variables indicates that these practices are perceived as interconnected components of a holistic experience, which collectively enhance patients’ psychological well-being. Additionally, the Kruskal–Wallis test further supported the presence of variability in patient experiences, highlighting significant differences in responses. This finding underscores the importance of considering how different aspects of treatment, including technological communication, shape the overall perception of the IVF experience.

### 4.7. Implications for Clinical Practice

The results of this study offer relevant insights for clinical practice in the field of assisted reproduction. The integration of techniques such as ultrasound-guided embryo transfer and real-time embryo visualisation appears to contribute positively to patients’ psychological well-being by reducing anxiety, enhancing satisfaction, and fostering a greater sense of involvement in the treatment process. These patient-centred interventions, although not directly linked to improved clinical outcomes in this study, may nonetheless support a more holistic and empathetic care model. Equally important is the continuity of care with the same physician throughout the IVF pathway, which emerged as a key determinant of emotional comfort and trust. Strengthening this relational aspect may help counteract the depersonalisation often perceived in fertility treatments, thereby improving the overall quality of care and patient adherence. However, the broader implementation of these practices may be challenged by practical barriers such as resource availability, organisational constraints, and the need for additional staff training. Despite these limitations, adopting strategies that prioritise visual engagement and relational continuity could enhance the patient experience. Future studies should investigate whether the perceived psychological benefits translate into measurable outcomes, employing multicentre designs and validated psychometric instruments, as well as objective markers of stress and emotional distress.

### 4.8. Limitations of This Study

This study has several limitations. It relies on self-reported perceptions from a single-centre cohort, without objective measures of psychological stress or well-being. As such, findings should be interpreted as reflective of subjective experiences rather than demonstrable clinical effects. Future research employing validated psychometric tools and multicentre designs is needed to assess potential psychological or clinical impacts more rigorously. It should be noted that the questionnaire was internally developed for the specific aims of this study and lacks external validation against established psychometric scales. This limitation may impact the robustness and external validity of the findings and should be acknowledged in their interpretation.

## 5. Conclusions

This descriptive study highlights the value patients place on certain aspects of their IVF treatment experience, particularly embryo visualisation and ultrasound-guided transfer. Patients reported perceiving greater calmness and a sense of security during these procedures. Additionally, the continuity of care with the same physician was perceived as an important factor in fostering trust and emotional comfort. While no clinical measures of stress or well-being were used, and no causal relationships can be inferred, these findings provide valuable insights into patient preferences and subjective experiences. Incorporating such elements into IVF protocols may enhance patient satisfaction and engagement with care.

## Figures and Tables

**Table 1:** Epidemiological characteristics of the groups.

	Study Group	Control Group	Student’s *t*-Test
**N°**	200	3174	
**Mean Age**	35.27	35.55	n.s.
**Marital Status**			
Married	64.7%	63.5%	n.s.
Cohabiting	35.3%	36.5%	n.s.
**Duration of Infertility**	2.3	2.5	n.s.
**Education Level**			
Primary School	3%	3.3%	n.s.
Secondary School	17%	16.8%	n.s.
High School	53.9%	53.4%	n.s.
University Degree	26.1%	26.5%	n.s.
**Occupation**			
Employed / Worker	37.5%	36.9%	n.s.
Housewife	24.7%	25.4%	n.s.
Professional	21.7%	20.9%	n.s.
Entrepreneur	9.9%	10.0%	n.s.
Unemployed	5.7%	6.1%	n.s.
Student	0.6%	0.7%	n.s.

n.s.= not significative.

**Table 2:** Descriptive analysis of responses.

Question	Likert Scale	Responses	%	Mean	Median	SD	Chi-Square
1. Is it better to interact always with the same doctor rather than with a different one each time?	1. Very much 2. Quite 3. Moderately 4. A little 5. Not at all	168 21 2 3 6	84 10.5 1 1.5 3	1.29	1	0.824	*143.1,* *p < 0.0001*
2. Are you more satisfied if the gynaecologist or embryologist shows you the images of the embryos to be transferred in real time during the transfer procedure?	1. Very much 2. Quite 3. Moderately 4. A little 5. Not at all	170 20 10 0 0	85 10 5 0 0	1.2	1	0.511	*167.1,* *p < 0.0001*
3. Does the ultrasound during the guided embryo transfer cause discomfort?	1. Very much 2. Quite 3. Moderately 4. A little 5. Not at all	5 2 6 15 172	2.5 1 3 7.5 86	1.265	1	0.786	*180.3,* *p < 0.0001*
4. Does the use of ultrasound during the embryo transfer procedure make you feel more at ease?	1. Very much 2. Quite 3. Moderately 4. A little 5. Not at all	170 16 9 1 4	85 8 4.5 0.5 2	1.265	1	0.747	*143.1,* *p < 0.0001*
5. Do you feel that viewing the embryos along with ultrasound during the transfer helps you face the procedure with more calmness?	1. Very much 2. Quite 3. Moderately 4. A little 5. Not at all	183 15 1 1 0	91.5 7.5 0.5 0.5 0	1.075	1	0.264	*176.9,* *p < 0.0001*
6. Do you feel that the ultrasound and viewing your embryos during the transfer could violate your privacy?	1. Very much 2. Quite 3. Moderately 4. A little 5. Not at all	0 0 2 1 197	0 0 1 0.5 98.5	1.005	1	0.070	*255.3,* *p < 0.0001*
7. Do you consider it important that your partner is present during the transfer?	1. Very much 2. Quite 3. Moderately 4. A little 5. Not at all	119 9 2 13 57	59.5 4.5 1 6.5 28.5	1.05	1	0.240	*110.0,* *p < 0.0001*
8. In the transfer room, does the presence of the midwife (other than your husband) interfere with your privacy?	1. Very much 2. Quite 3. Moderately 4. A little 5. Not at all	1 2 0 3 194	0.5 1 0 1.5 97	1.025	1	0.156	*244.7,* *p < 0.0001*

*p* < 0.0001 significative.

**Table 3:** Correlation analysis between questions and significance.

Spearman r	Question 2	Question 4	Question 5
**r**	0.007315	0.01406	0.03239
**95% confidence interval**	−0.1356 to 0.1499	−0.1290 to 0.1565	−0.1109 to 0.1744
***p* value**			
***p* (two-tailed)**	0.918	0.843	0.649
***p* value summary**	ns	ns	ns
**Exact or approximate *p* value?**	Approximate	Approximate	Approximate
**Significant? (alpha = 0.05)**	No	No	No
**Number of XY pairs**	200	200	200

**Table 4:** Analysis of Differences between responses to multiple questions.

Kruskal–Wallis Test	*p*-Value	Approximate *p*-Value?	Medians Vary Significantly (*p* < 0.05)?	Number of Groups	Kruskal–Wallis Statistic
	<0.0001	Approximate	Yes	8	67.99

*p* < 0.0001 significative.

**Table 5:** Exploratory factor analysis. Total folded variance.

Question	Initial Eigenvalues			Loading Sums of Extraction Squares		
	Total	% of Variance	% Cumulative	Total	% of Variance	% Cumulative
1	1.296	16.195	16.195	1.296	16.195	16.195
2	1.126	14.073	30.267	1.126	14.073	30.267
3	1.051	13.136	43.404	1.051	13.136	43.404
4	1.024	12.797	56.201	1.024	12.797	56.201
5	0.967	12.087	68.288			
6	0.943	11.788	80.075			
7	0.884	11.053	91.129			
8	0.710	8.871	100.000			

Extraction method: principal component analysis.

## Data Availability

The datasets analysed during the current study are available from the corresponding author on reasonable request. It will be possible to request data up to 5 years from the publication of this research.

## References

[1] Verhaak C.M., Smeenk J.M., Evers A.W., Kremer J.A., Kraaimaat F.W. (2020). Psychological outcomes in women undergoing fertility treatment: A systematic literature review. Hum. Reprod. Update.

[2] Eugster A., Heier H., Kjær T.M., Madsen L.B. (2022). Mental health in women undergoing in vitro fertilisation: A longitudinal analysis. J. Psychosom. Obstet. Gynaecol..

[3] Cousineau T.M., Domar A.D., Sabini E.A. (2022). Stress and coping in women undergoing in vitro fertilisation: A meta-analysis. J. Assist. Reprod. Genet..

[4] Boivin J., Bunting L., Collins J. (2021). Psychological and relational impacts of infertility and assisted reproduction: An update. Fertil. Steril..

[5] Mac Dougall K., Wilschut J., van de Wiel H. (2023). Psychological distress in fertility treatment patients: A cross-sectional study. BMC Women’s Health.

[6] Bengoa R., Kawar R., Key P., Leatherman S., Massoud R., Saturno P. (2006). Quality of Care: A Process for Making Strategic Choices in Health Systems.

[7] Peddie V., Fishel S. (2018). Patient-centred care in fertility treatment: A critical review. Hum. Reprod..

[8] Klemetti R., Raitanen J. (2020). The importance of patient-centred communication in fertility treatment: A systematic literature review. Eur. J. Obstet. Gynecol. Reprod. Biol..

[9] Gleicher N., Barad D.H. (2017). The role of patient-centred care in infertility treatment outcomes. J. Assist. Reprod. Genet..

[10] Fleming L., Millar A. (2019). Reframing fertility care: The centrality of patient empowerment and choice. Reprod. Health.

[11] Hammarberg K., McDonald H. (2021). Women's experiences of infertility and assisted reproductive technologies: A patient-centred perspective. BMC Women’s Health.

[12] Cousineau T.M., Domar A.D. (2020). Psychological aspects of fertility treatment: The importance of patient-centered care. Fertil. Steril..

[13] Tuil W.S., ten Hoopen A.J., Braat D.D., de Vries Robbe P.F., Kremer J.A. (2006). Patient-centred care: Using online personal medical records in IVF practice. Hum. Reprod..

[14] Mourad S.M., Nelen W.L.D.M., Akkermans R.P., Vollebergh J.H., Grol R.P., Hermens R.P., Kremer J.A. (2010). Determinants of patients’ experiences and satisfaction with fertility care. Fertil. Steril..

[15] Verberg M.F.G., Eijkemans M.J.C., Heijnen E.M.E.W., Broekmans F.J., de Klerk C., Fauser B.C.J.M., Macklon N.S. (2008). Why do couples drop out of IVF treatment? A prospective cohort study. Hum. Reprod..

[16] Raoul J., Nargund G., Lisi F. (2019). Ultrasound-guided embryo transfer in assisted reproduction: A systematic review and meta-analysis. Hum. Reprod. Update.

[17] Malvasi A., Damiani G.R., Edoardo D.N., Vitagliano A., Dellino M., Achiron R., Ioannis K., Vimercati A., Gaetani M., Cicinelli E. (2023). Intrapartum ultrasound and mother acceptance: A study with informed consent and questionnaire. Eur. J. Obstet. Gynecol. Reprod. Biol. X.

[18] Souter V.L., Penney G., Hopton J.L., Templeton A.A. (1998). Patient satisfaction with the management of infertility. Hum. Reprod..

[19] Dancet E.A., Nelen W.L., Sermeus W., De Leeuw L., Kremer J.A., D‘Hooghe T.M. (2010). The patients’ perspective on fertility care: A systematic review. Hum. Reprod. Update.

[20] Van Empel I.W., Nelen W.L., Tepe E.T., van Laarhoven E.A., Verhaak C.M., Kremer J.A. (2010). Weaknesses, strengths and needs in fertility care according to patients. Hum. Reprod..

[21] Cousineau T.M., Domar A.D. (2007). Psychological impact of infertility. Best Pract. Res. Clin. Obstet. Gynaecol..

[22] Young G.J., Meterko M., Desai K.R. (2000). Patient satisfaction with hospital care: Effects of demographic and institutional characteristics. Med. Care..

[23] Hammarberg K., Astbury J., Baker H. (2001). Women‘s experience of IVF: A follow-up study. Hum. Reprod..

[24] Crow R., Gage H., Hampson S., Hart J., Kimber A., Storey L., Thomas H. (2002). The measurement of satisfaction with healthcare: Implications for practice from a systematic literature review. Health. Technol. Assess..

[25] Schmidt L., Holstein B.E., Boivin J., Sangren H., Tjornhoj-Thomsen T., Blaabjerg J., Hald F., Andersen A.N., Rasmussen P.E. (2003). Patients’ attitudes to medical and psychosocial aspects of care in fertility clinics: Findings from the Copenhagen Multi-centre Psychosocial Infertility (COMPI) Research Programme. Hum. Reprod..

[26] Di B.Z., Harkness E., Ernst E., Georgiou A., Kleijnen J. (2001). Influence of context effects on health outcomes: A systematic review. Lancet.

[27] Redshaw M., Hockley C., Davidson L.L. (2007). A qualitative study of the experience of treatment for infertility among women who successfully became pregnant. Hum. Reprod..

[28] Gabel L.L., Lucas J.B., Westbury R.C. (1993). Why do patients continue to see the same physician?. Fam. Pract. Res. J..

[29] Saultz J.W., Albedaiwi W. (2004). Interpersonal continuity of care and patient satisfaction: A critical review. Ann. Fam. Med..

[30] Pereira Gray D.J., Sidaway-Lee K., White E., Thorne A., Evans P.H. (2018). Continuity of care with doctors-a matter of life and death? A systematic review of continuity of care and mortality. BMJ Open.

[31] Robinson J.P., Shaver P.R., Wrightsman L.S. (1991). Measures of Personality and Social Psychological Attitudes.

[32] James C.A. (1992). The nursing role in assisted reproductive technologies. NAACOG's Clin. Issues Perinat. Women's Health Nurs..

[33] Mitchell A., Mittelstaedt M.E., Wagner C. (2005). A survey of nurses who practice in infertility settings. J. Obstet. Gynecol. Neonatal Nurs..

[34] Zivaridelavar M., Kazemi A., Kheirabadi G.R. (2016). The effect of assisted reproduction treatment on mental health in fertile women. J. Educ. Health Promot..

[35] Schmidt L., Holstein B.E., Christensen U., Boivin J. (2003). The impact of infertility on quality of life and mental health. Hum. Reprod..

[36] Balen A.H., Hiam C., Ruiter L. (2015). Psychological outcomes in women undergoing fertility treatment. Cochrane Database Syst. Rev..

[37] Ludwig M., Diedrich K., Langen D. (2012). The impact of IVF on the psychological well-being of women. Reprod. Biomed. Online.

[38] Hart R., Doherty M. (2013). Fertility treatment and emotional stress: A systematic literature review. J. Psychosom. Obstet. Gynaecol..

[39] Stanhiser J., Steiner A.Z. (2018). Psychosocial Aspects of Fertility and Assisted Reproductive Technology. Obstet. Gynecol. Clin..

[40] Ayling K., Fairclough L., Tighe P., Todd I., Halliday V., Garibaldi J., Royal S., Hamed A., Buchanan H., Vedhara K. (2018). Positive mood on the day of influenza vaccination predicts vaccine effectiveness: A prospective observational cohort study. Brain Behav. Immun..

[41] Reis H.T., Clark M.S., Pereira Gray D., Tsai F.F., Brown J.B., Stewart M., Underwood L.G. (2008). Measuring responsiveness in the therapeutic relationship: A patient perspective. Basic Appl. Soc. Psych..

[42] Cozzolino M., Vitagliano A., Di Giovanni M.V., Laganà A.S., Vitale S.G., Blaganje M., Drusany Starič K., Borut K., Patrelli T.S., Noventa M. (2018). Ultrasound-guided embryo transfer: Summary of the evidence and new perspectives. A systematic review and meta-analysis. Reprod. Biomed. Online.

[43] Teixeira D.M., Dassunção L.A., Vieira C.V., Barbosa M.A., Coelho Neto M.A., Nastri C.O., Martins W.P. (2015). Ultrasound guidance during embryo transfer: A systematic review and meta-analysis of randomized controlled trials. Ultrasound Obstet. Gynecol..

[44] Larue L., Keromnes G., Massari A., Roche C., Moulin J., Gronier H., Bouret D., Cassuto N.G., Ayel J.P. (2017). Transvaginal ultra-sound-guided embryo transfer in IVF. J. Gynecol. Obstet. Hum. Reprod..

